# Potential therapeutic targets of Guggulsterone in cancer

**DOI:** 10.1186/s12986-017-0180-8

**Published:** 2017-02-28

**Authors:** Ajaz A. Bhat, Kirti S. Prabhu, Shilpa Kuttikrishnan, Roopesh Krishnankutty, Jayaprakash Babu, Ramzi M. Mohammad, Shahab Uddin

**Affiliations:** 1 Translational Research Institute, Hamad Medical Corporation, PO Box 3050, Doha, Qatar; 2 Department of Biochemistry and Molecular Biology, University of Nebraska Medical Center, Omaha, NE USA

**Keywords:** Natural compounds, Guggulsterone, Cancers, Chemoprevention, Molecular targets

## Abstract

Natural compounds capable of inducing apoptosis in cancer cells have always been of considerable interest as potential anti-cancer agents. Many such compounds are under screening and development with their potential evolution as a clinical drug benefiting many of the cancer patients. Guggulsterone (GS), a phytosterol isolated gum resin of the tree *Commiphora mukul* has been widely used in Indian traditional medicine as a remedy for various diseses. GS has been shown to possess cancer chemopreventive and therapeutic potential as established by in vitro and in vivo studies. GS has been shown to target constitutively activated survival pathways such as PI3-kinase/AKT, JAK/STAT, and NFκB signaling pathways that are involved in the regulation of growth and inflammatory responses *via* regulation of antiapoptotic and inflammatory genes. The current review focuses on the molecular targets of GS, cellular responses, and the animal model studies in various cancers. The mechanistic action of GS in different types of cancers also forms a part of this review. The perspective of translating this natural compound into a clinically approved drug with its pros and cons is also discussed.

## Background

**Fig. 1:**
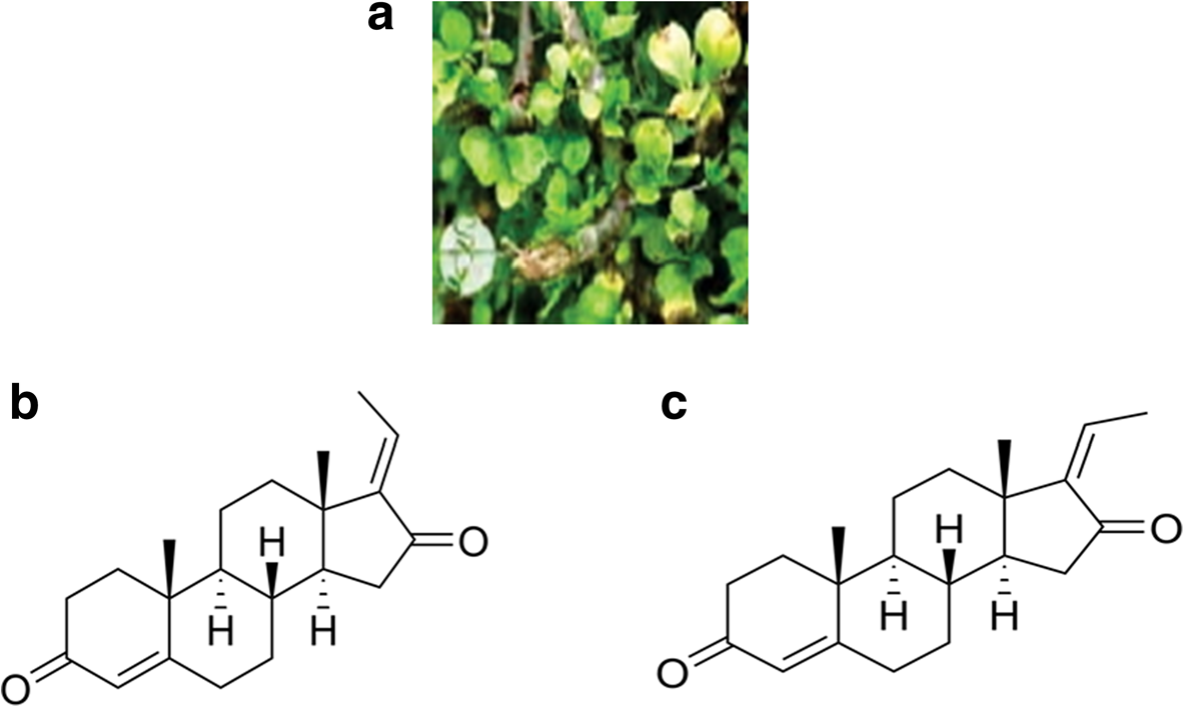
**a** The Plant *Commiphora mukul*. The chemical structure of Guggulsterone isoforms, E-Guggulsterone (**b**) and Z-Guggulsterone (**c**)

The important aspect in cancer chemoprevention is to identify drugs or compounds that kill the tumor cells with lower toxicity to healthy tissue. The other alternative approach could be to identify agents, which either slows or even halts the tumor progression. The growing evidence linked the deregulation of apoptosis in cancer cells and supports the hypothesis that targeting deregulated apoptotic signaling pathways could serve as a tool for cancer prevention [1, 2]. Interestingly, the chemotherapeutic drugs that are currently used exert their cytotoxic effect through the induction of apoptosis [3–5]. Therefore, the success of cancer therapy depends on the sensitivity of cancer cells to respond to the therapeutic agents that turn on the apoptotic process. The signaling cascade that leads to apoptosis can be induced by a vast variety of drugs with diverse chemical structures and different mechanisms of action, notably through the inhibition of survival and increased expression of pro-apoptotic genes. Natural compounds have been shown to have less cytotoxicity and efficient in blocking prosurvival/ growth signal transduction pathways. Interestingly, most of the natural compounds currently available as pharmaceutical products isolated from plant extracts have proven to exhibit anti-tumor properties in vivo and in vitro. One such traditional medicine, guggulsterone (GS), [4, 17(20)-pregnadiene- 3, 16-dione], a plant polyphenol extracted from the gum resin of the Commiphora mukul tree has been broadly used for centuries to treat multiple human diseases [6–8]. The active ingredients found in the extract from the gum resin of Mukul tree are the isomers E- and Z-GS (Fig. 1) and both these forms have been extensively used to treat multiple disorders. It has been shown that GS is an antagonist of bile acid farnesoid X receptor (FXR) [9–11] and inhibition of FXR expression by GS causes anticancer activity in many cancer cells [12–17]. There is also accumulating evidence about the role of GS in cholesterol homeostasis regulation by increasing the transcription of bile salt export pump [10, 11, 17]. GS has been shown to play an important role in nutritional metabolism as it has been found to inhibit cholesterol synthesis in the liver *via* antagonism of the FXR and the bile-acid receptor [18]. GS has been widely used for the treatment of hyperlipidemia in humans [5, 19]. A number of studies have demonstrated that GS efficiently decreases low density lipoprotein cholesterol and triglyceride levels in serum and increases high density lipoprotein cholesterol levels [20, 21]. Specifically, E and Z isoforms of GS have been identified as active ingredients for lipid-lowering [22]. GS has been shown to bind FXR and prevent the expression of FXR agonist-mediated genes [8, 23]. Furthermore, it has been demonstrated that the lipid lowering effect of GS in liver are due to inhibition of FXR as confirmed from FXR knockout mice studies [8].

**Fig. 2:**
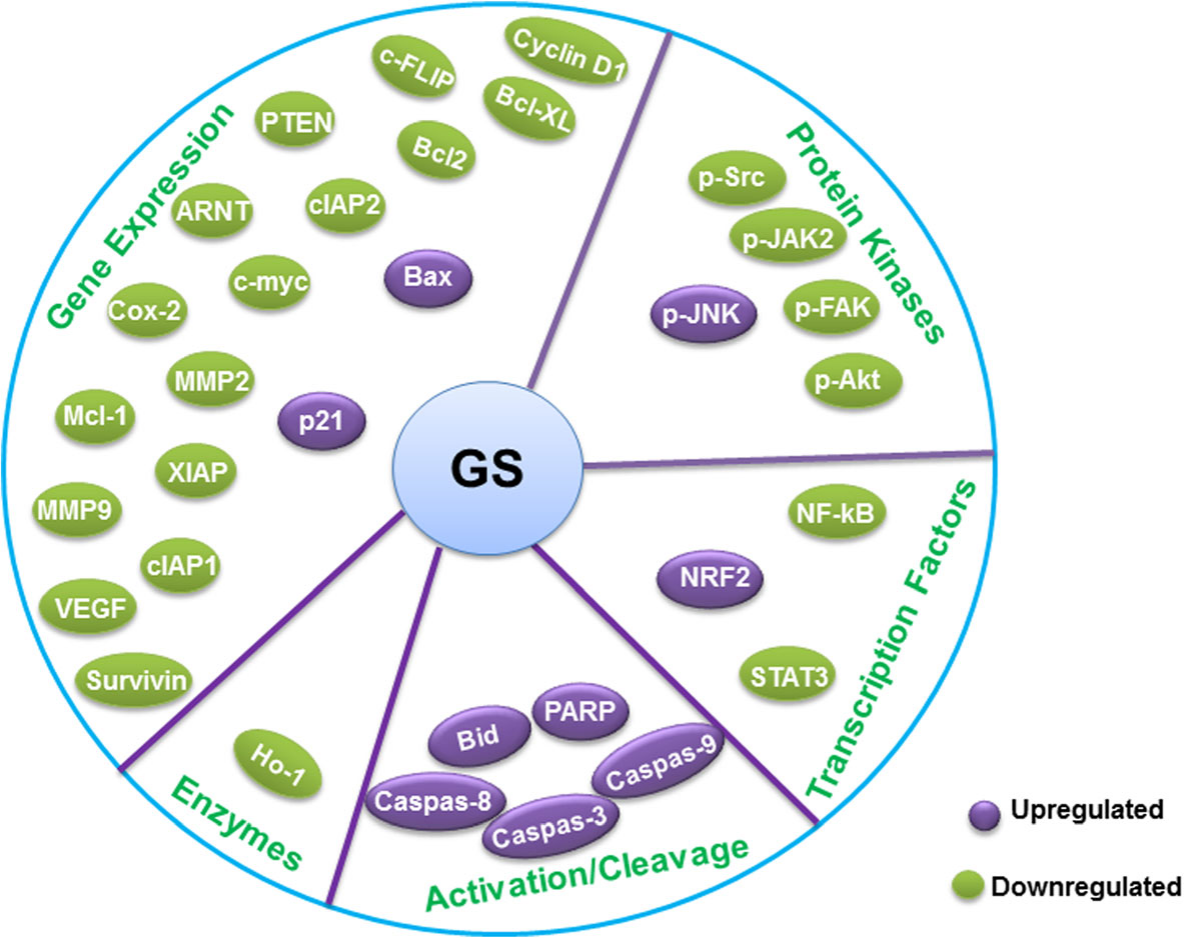
Biochemical and molecular targets of Guggulsterone. Guggulsterone exerts anti-cancer effects through activation or suppression of protein kinases, transcription factors, anti-oxidant enzymes, cell cycle regulators, proapoptotic and antiapoptotic proteins. GS exerts anti-inflammatory effects through suppression of nuclear factor-kB (NF-kB), which plays a crucial role in the inflammatory processes by regulating the expression of diverse proinflammatory proteins, including cyclooxygenase-2 (COX-2). GS fortifies cellular defense against oxidative stress by inducing the *de novo* synthesis of the powerful antioxidant enzyme heme oxygenase-1 (HO-1). GS induces apoptosis by increasing the expression of proapoptotic proteins while decreasing the levels of antiapoptotic proteins (e.g., IAP1, XIAP, Bfl-1/A1, Bcl-2, cFLIP, Survivin, etc.). GS induces apoptosis by increasing the expression of proapoptotic proteins while decreasing the levels of antiapoptotic proteins (e.g., IAP1, XIAP, Bfl-1/A1, Bcl-2, cFLIP, Survivin, etc.). GS suppresses invasion and metastasis by targeting MMPs, FXR etc

GS has been found to induce apoptotic cell death in many types of cancer [24–28] *via* activation of caspases, increased expression of genes of Bcl-2 family members and generation of reactive oxygen intermediates. A number of studies have shown that GS strongly inhibits the activation of various survival signaling pathways including, PI3-kinase/AKT, JAK/STAT and nuclear factor-kB (NF-kB) in various cancer cells [29–31] (Table 1). Constitutive activity of NF-kB plays a crucial role in growth and proliferation of malignant cells *via* regulating expression of several antiapoptotic genes. GS was found to efficiently suppress the expression of these antiapoptotic genes in many cancer cells (Fig. 2). In addition, GS has also been shown to suppress the ionizing radiation (IR)-mediated activation of NF-κB and augments the radiosensitivity of human cancer cell lines [32]. Further, GS is reported to reduce cell growth as well as prevents IR-induced DNA damage repair [32] and GS has been shown to induce apoptosis in a wide rangeof cancer cells [24, 25, 27, 28, 33–36]. The detailed molecular targets of GS and mechanisms regulating apoptosis in various cancers are discussed in this review.

**Table 1:** Anticancer activity of GS in in vitro experimental model and underlying molecular targets

Cancer Type	Model/System	Molecular Targets	References
Pancreatic cancer	Human pancreatic cancer cell lines	↓FXR reduced ↓ NF-*κ*B, ↓Cyclin D1, ↓Bcl-2, ↓XIAP↓MPP9, ↓STAT3, ↓FAK, ↓Src, p-AKT,c-June, ↑Caspase-3,↑Bax	[33, 38, 39]
Head and Neck cancer	Head and neck carcinoma cell line	↓ PI3-kinase/AKT, ↑Bax, ↑Bad	[88]
Esophagael cancer	Esophageal adenocarcinoma cell lines	↓caudal type homeobox 2,↓Cox2,↓NFkB, ↓FXR, ↓ RAR-β2, ↑caspase-8,caspase-9,caspase-3	[47, 48]
Colon cancer	Colon cancer cell line	↓cIAP-1, ↓cIAP-2, ↓Bcl-2, ↓STAT3, ↓VEGF, ↑truncated Bid, ↑Fas, ↑p-JNK, ↑p-c-Jun	[50, 51]
Breast cancer	Breast cancer cell lines	↓cyclin D1, ↓C-myc, ↓survivin, ↓TCF-4, ↓IKK/NF-κB, ↓MAPK/AP-1, ↓MMP-9 ↓	[34, 89]
Prostate cancer	Prostate cancer cell lines	↑caspase-9, ↑caspase-8, ↑caspase-3, ↑Bax **↓**Bcl-2 and **↓**Bcl-xL	[27]
Hepatocellular carcinoma	Hepatocellular carcinoma cell lines	**↓**TGF-β1, **↓**VEGF,**↓** Bcl-2,↑Bax, **↓** NF-κB, **↓**STAT3	[83, 85]
Hematological malignancies	Leukemic cell lines	↓Bfl-1/A1, ↓XIAP, ↓cFLIP, ↓Bcl-2, ↓BclXL, ↓survivin ↑caspase 8, ↑bid cleavage, ↑cytochrome c release, ↑caspase 9, ↑ caspase 3, ↑ PARP cleavage.	[25]

## Guggulsterone and cancer

Since several decades extensive research has revealed that many chronic illnesses are caused by the deregulation of multiple genes mainly involved in cell cycle control enabling the cells to divide uncontrollably leading to metastasis [1–4]. Most of the conventional drugs primarily target a single gene product or signaling pathway at a given time, thus having a limited scope for the treatment. In addition these medicines exhibit many toxic side effects. Due to these limitations, there is a growing trend towards alternative medicines such as traditional medicine derived from natural compounds which are safe and have broad spectrum activity [37]. GS is one such ancient medicine that targets multiple signaling molecules with a varied range of mechanisms with its proven antiproliferative and proapoptotic effects in vitro *and* in vivo *studies* (Tables 1 and 2). The following sections describe GS-mediated anticancer effects and its potential targets in various cancers.

**Table 2:** Anticancer activity of GS in in vivo animal experimental models

Cancer Type	Model/System	Antitumor effects	References
Pancreatic cancer	Pancreatic cancer cell line xenograft tumors	↓ tumor size	[39]
Colon Cancer	HT-29 xenograft tumors	↓ tumor size	[50]
Esophageal cancer	Esophageal adenocarcinoma cell lines	↓ tumor size	[47]
Breast cancer	MCF7 xenograft tumors	↓Bcl-2 and P-glycoprotein expression ↑Chemosensivity of doxorubicin in vivo	[75]
Prostate cancer	DU145 prostate cancer cells implanted in mouse	↓ tumor size, ↓ angiogenesis↓ VEGFR-2	[28]

## Pancreatic cancer

Pancreatic tumors are highly aggressive, and there is an urgent need of therapeutic strategy for the better management of this cancer. The existing chemotherapeutic drugs cause high toxicity and drug resistance. Macha et al. [33] using in vitro model have shown that GS prevents cell proliferation, inhibits cell motility reduces cell invasion and induces apoptotic cell death in many pancreatic cancer cell lines. The anti-cancer activity of GS was correlated with the down-regulation of anti-apoptotic proteins, cell cycle progression proteins and up-regulation of proapoptotic proteins. Furthermore, the reduced motility and suppressive effects on invasion in pancreatic cancer cells by GS were associated with the disruption of cytoskeletal organization, inhibition of FAK and Src kinase signaling. GS treatment was also found to reduce mucin MUC4 gene expression by inhibition of JAK kinase mediated signaling [33]. A recent study using pancreatic cancer cell lines has reported a significant reduction in cell migration and invasion by GS-mediated FXR inhibition indicating the potential role of FXR overexpression in lymphatic metastasis of pancreatic cancer [38].

GS has been found to increase the sensitivity of conventional chemotherapeutic agents such as gemcitabine. Treatment of pancreatic cancer cells with GS augmented apoptotic cell death when combined with gemcitabine as compared to treatment either with gemcitabine or GS alone. In addition, the tumors from xenografted mice in vivo model showed a better antitumor response to GS combination treatment compared to gemcitabine or GS alone.

Studies from in vitro and in vivo (Tables 1 and 2) settings have shown that the combination treatment leads to increased growth inhibition as well as apoptosis through a cascade of events involving the down-regulation of NF-κB, inhibition of AKT activity, downregulation of antiapoptotic gene BcL-2, upregulation of proapoptotic gene Bax and, activation of c-Jun NH(2)-terminal kinase (JNK) [39].

## Head and neck cancer

A number of studies provide substantial evidence that GS suppressed the growth/or induced apoptotic cell death of head and neck squamous cell carcinoma (HNSCCC) [40–42]. GS-mediated inhibition of HNSCC proliferation is caused by inactivation of NF-κB and STAT3 signaling cascades. GS treatment of HNSCC cells prevents NF-κB activation and leads to its degradation resulting in the inhibition of inflammatory and angiogenic responses as well as progression and metastasis [35, 36]. GS was also able to inhibit COX-2 and vascular endothelial growth factor (VEGF) which contributes to inflammation and angiogenesis [35]. The antiproliferative and pro-apoptotic action of GS in SCC4 cells has been due to downregulation of various antiapoptotic genes including Bcl-2, XIAP, Mcl1, survivin, cyclin D1 and c-myc. Furthermore GS-mediated downregulation of these genes resulted sequential activation of caspas-9,-3 and cleavage, of poly-ADP-ribose phosphate (PARP) [25, 43].

Recently it has been reported that combination of GS and bortezomib, a proteasome inhibitor synergize the inhibition of signaling molecules that are essential for the proliferation and survival of malignant cells [44, 45]. These reports further suggest that co-treatment of HNSCC cells with bortezomib and GS potentiated effects on cell death and inhibited clonogenic survival. These findings correlated with inhibition of NF-κB signaling pathway [44] and induction of the proapoptotic proteins Bik, Bim, and Noxa [45]. These results suggest that GS could significantly improve the therapeutic activity of bortezomib against HNSCC in cotreatment strategy. Similar studies with erlotinib, cetuximab, and cisplatin in HNSCC cell lines further supported the synergistic activity of GS towards enhanced efficacy to apoptosis, cell cycle arrest and invasion [46].

## Esophageal adenocarcinoma

Chronic Gastroesophageal Reflux Disease (GERD) is the main risk factor for the development of Barrett's esophagus (BE) and its progression to esophageal adenocarcinoma (EAC). Studies have shown the significant overexpression of bile acid receptor FXR in Barrett's esophagus and treatment of Barrett's esophagus-derived cell line with GS was found to significantly reduce the expression of FXR.. Treatment of Barrett's esophagus-derived cell line with GS was linked with a significant increase in the percentage of apoptotic cells and of the caspase -3 activity signifying that FXR may contribute to the regulation of apoptosis [12]. A similar study further showed that the inhibition of FXR in esophageal adenocarcinoma tissues either with an expression of FXR shRNA or treatment with GS suppressed tumor cell viability and induced apoptosis in vitro and in vivo [16] suggesting GS as a potential antagonist to FXR overexpression and a cancer chemotherapeutic. GS has been shown to have additive effect in suppressing esophageal cancer cell growth in vitro and in nude mouse xenografts when combined with amiloride and this activity is due to the inhibition of gastric acid-inducing gene Na + /H + exchanger-1 (NHE-1) [47]. In another study, GS has been shown to suppress bile acid induced caudal type homeobox 2 (CDX2) and cyclooxygenase-2 (COX-2) expression, which are critical in the development of Barrett’s Esophagus and esophageal adenocarcinoma and this effect is due to inhibition of NF-kB activity [48].

## Colon cancer

Although there is a major progress in the advancement of chemotherapeutic agents for the treatment of colon cancer, however, the relapse rates still remain to be elevated for the existing drugs. The current chemotherapy has been benefecial only to the patients with advanced colorectal cancerregarding their survival and quality of life [49]. The other problems that happen to be with the conventional chemotherapy are systemic side effects and increased chemoresistance. Because of these limitations, there is an urgent need for more efficient and safer therapeutic strategies for advanced or untreatable colorectal cancer. Interestingly, a considerable amount of attention towards the anti-tumor properties of various phytochemicals has developed in the last few years both in vivo and in vitro. In connection to this, GS was found to possess potential anti-tumor effects in several cancer cell types including colorectal cancer [24–28, 33]. Emerging studies have also demonstrated that GS and its derivatives significantly reduced dextran sulfate sodium (DSS)-induced acute murine colitis. Further, more this effect was found due to GS-mediated down-regulation of NF-kB signaling pathway. These findings suggest that GS- mediated targeting of signaling pathways may be an attractive strategy for the treatment of inflammatory bowel disease (IBD) [30]. GS has also been shown to induce apoptotic cell death via activation of caspase cascade, downregulation of inhibitor of apoptosis proteins Bcl-2, and activation of JNK kinase [50]. GS treatment of colon cancer cell lines has been shown to block angiogenesis and metastasis by inactivation of STAT3 activity and downregulation of VEGF expression [51].

Bile acid receptor FXR besides playing a role in lipid metabolism, has also been shown to play an important role in intestinal carcinogenesis. Reduced expression of FXR mRNA has been found in human colon polyps and even more pronounced in colon adenocarcinomas [52, 53]. FXR has been shown to suppress intestinal tumorigenesis in both the *Apc* ^*Min+/−*^ and chronic colitis mouse models of intestinal neoplasia by regulating Wnt signaling and apoptosis [54]. FXR-deficient mice have been shown to exhibit increased intestinal epithelial cell proliferation and tumor development [55]. Recently, Peng et al. [15] have demonstrated that treatment of colon cancer cell lines with FXR antagonist GS or FXR siRNA lead to phosphorylation of EGFR and ERK whereas treatment with GW4064 or FXR overexpression prevented cell proliferation by dephosphorylation of EGFR and ERK. In addition treatment of colon cancer cell lines with GS and GW4064 also caused dose-dependent changes in Src (Tyr416) phosphorylation.

## Breast cancer

Advanced early screening as well as detection methods have been developed, and several are under development for various cancers, including breast cancer. However, breast cancer continues to be the most challenging owing to its high frequency among women worldwide [56, 57]. A number of studies have suggested that various mechanisms are responsible for the incidence and development of breast carcinogenesis. NF-κB pathway is highly activated in breast cancer [58, 59]. NF-κB is one of the key molecules shown to regulate the expression of various antiapoptotic genes that plays a crucial role in tumorigenesis [29, 60]. These genes are *bcl-2*, and *bcl-xl*, adhesion molecule encoding genes, chemokines, and inflammatory cytokines; and cell cycle regulators. Therefore, targeting NF-κB and its associated partners could be an important therapeutic strategy for the management of breast cancer. Previously it has been demonstrated that GS inhibits NF-κB activation *via* IκBα kinase suppression, along with the dephosphorylation and degradation of IκBα. Moreover, GS also interferes nuclear translocation of p65 and NF-κB- mediated reporter gene activity [29]. GS treatment of cancer cells has been found to abrogate the expression of NF-κB-mediated antiapoptotic genes, as well the genes involved in regulation of inflammation and tumor metastasis [29–31]. Activation of pro-survival signaling pathways play a crucial role in suppression of apoptotic cell death during radiosensitization of cancer cells [61]. Ionizing radiation (IR) has been found to activate NF-κB and associated pathways that are involved in growth and survival of cancer cells [62]. Interestingly, GS treatment abrogated the IR-mediated activation of NF-κB and augmented the radiosensitivity of human tumor cell lines. Expression of hormone receptors by breast cancer cells makes them sensitive to hormonal therapy. However, the use of ER antagonists is restricted due to unwanted side effects [58]. Therefore, the development of new, safe and affordable therapeutics against ER-breast cancer cells harboring ER as well is much needed. GS has been found to downregulate the expression of ERα in breast cancer cells implicating that it could be a viable therapeutic useful in the treatment of ER-positive tumors that are resistant to tamoxifen [32].

It has been shown that the isomer of GS, *cis*-GS prevented TPA-upregulated MMP expression via obstructing IKK/NF-kB signaling. On the other hand, *trans*-GS was found to inhibit MAPK/AP-1 signaling pathway in MCF7 breast cancer cell line. Furthermore, co-treatment of breast cancer cells with these isomers exhibited additive effects on the inhibition of cell invasion. Another key signaling in growth and development of tumors is the Wnt/β-Catenin and its associated pathways [34]. This pathway has been shown to play a significant role in the initiation, progression, and metastasis of breast cancers [63–65]. The Wnt signaling exerts its effects on TCF-mediated transcription *via* β-Catenin [63–66]. Therefore, intercepting the signaling between Wnt and β-Catenin may prove a better way in developing new cancer therapeutics. Research in this direction, using natural products have already been shown to be promising [67–69]. It has been demonstrated that the expression of c-Myc, cyclin D1, and survivin, downstream of Wnt/β-Catenin signaling, were inhibited by guggulipid (GL) and z-GS in breast cancer significantly. In addition, treatment with GL in human breast cancer cells results in downregulation of TCF-4 protein expression significantly.

DNA methylation, as well as histone modifications, are other attractive targets for therapeutics strategy for the management of cancer [70]. Deregulated methylation can result in silencing of various functional genes including tumor suppressors that often lead to cancer development and progression [70]. Inhibition of DNA methyltransferases has been shown to suppress tumor formation [71]. Interestingly, some epigenetic modifications that regulate normal cellular activity via dietary phytochemicals have proved to be reducing cancer susceptibility [72]. It has been demonstrated that GS treatment of breast cancer cells inhibits the expression of DNA (cytosine-5)-methyltransferase 1(DNMT1) and HDAC1 [73].

Current chemotherapy for breast cancer faces a major problem of drug resistance. A viable approach to avoid drugs causing drug resistance is by utilizing non-toxic compounds in combination with conventional chemotherapeutic agents. Xu et al*.* [74] have reported that multidrug resistance developed by the expression of P-glycoprotein in breast cancer cells (MCF/Dox) against doxorubicin can be improved by treatment with GS. Co-treatment of MCF/DOX cells with GS and doxorubicin results in a significant increase in chemosensitivity. A similar observation was found in xenografts generated from MCF-7/DOX cells [75]. BCRP/ABCG2, an ABC transporter is overexpressed in breast cancer cells and has been shown to be involved in multidrug resistance [76]. Combined treatment of GS with bexarotene a retinoid X receptor agonist resulted in cytotoxicity via downregulation of BCRP/ABCG2 in breast cancer cell line.

## Prostate cancer

Prostate cancer progression is a slow multistep process which begins with localized and low-grade lesions to high-grade and metastatic carcinomas resulting in a significant number of deaths in men [77, 78]. The slow progression and late diagnosis of prostate cancer allow a substantial opportunity for intervention to prevent this malignancy [79].

There are a considerable number of preclinical studies showing the anticancer activity of GS in prostate cancers. Singh and colleagues [24], have demonstrated that the GS treatment of human prostate cancer cell line, PC-3 resulted in efficient cytotoxic effects without affecting the normal prostate epithelial cell line (PrEC). In addition, GS-mediated growth inhibition of PC-3 cells occurs due to apoptosis rather than the cell cycle progression arrest. Furthermore, GS-induced apoptotic cell death correlated with the enhanced expression of Bcl-2 family members such as Bax and Bak and sequential activation of caspase cascade [27]. Furthermore, Xu and Sing [28] reported that z-GS treatment of DU145 implanted cells in mice angiogenesis *via* suppression of VEGF–VEGF-R2–Akt signaling axis. These findings were in accordance with the later reports in which treatment of GS was shown to downregulate the expression of antiapoptotic gene products including XIAP, survivin, cFLIP, Bcl-2, Bcl-Xl, c-myc and COX-2 [24, 25]. The mechanism by which GS-induced apoptosis in prostate cancer cells is not known, however, GS-mediated generation of ROS, which leads to activation of JNK has been implicated as one of the mechanisms leading to cell death in these cancer cells [24]. Treatment of LNCaP and PC3 cells with GS-causes the activation of JNK and p38. Interestingly, GS treatment activates extracellular signal-regulated kinase 1/2 (ERK1/2) only in LNCaP cells. GS treatment of prostate cancer cells resulted in the generation of ROS but not in normal PrEC prostate cells. In addition, PrEC cells showed resistance to GS-mediated activation of JNK kinase. Furthermore, overexpression of catalase and superoxide dismutase in prostate cancer cells prevented GS-mediated apoptosis and JNK activation [25]. In another study from the same group, treatment of prostate cancer cells, LNCaP withGL, a crude extract from which GS has been isolated, showed a dose-dependent inhibition of cell viability [80]. GL-mediated inhibition of cell viability in prostate cancer cells correlated with apoptosis as supported by an increase in cytoplasmic histone-associated DNA fragmentation and cleavage of PARP. Further, GL-induced apoptosis has been found to be associated with the generation of ROS and JNK activation along with the upregulation of proapoptotic proteins Bax and Bak and downregulation of Bcl-2 expression. During GS-mediated apoptosis, activation of JNK preceded before upregulation of Bax activation [80]. It was further shown that z-GS, another form of GS causes inhibition of angiogenesis *via* inactivation of AKT, and suppression of angiogenic factors such as VEGF and G-CSF [28]. The growth inhibitory effect of GS in prostate cancer has also been proposed to be due to inactivation of ATP citrate lyase (ACL or ACLY) which has been shown to exhibit crosstalk with the AKT signaling [81]. ACL is an extra-mitochondrial enzyme that has been demonstrated to play a crucial role in cellular lipogenesis, and its dysregulated expression is reported various cancers such as colon, prostate, liver, lung cancers as well as in many immortalized cells. Aberrant expression of ACL was reported to be inversely associated with tumor stage as well as differentiation and serves as a negative prognostic marker [82]. Thus, targeting ACL with GS in prostate cancermight be of potential therapeutic intervention strategy.

## Liver, lung and ovarian cancer

It is well-known fact that the growth inhibitory and proapoptotic effects of GS are mediated through various mechanisms and certain cancers share the common mechanism. In liver cancer, the mechanism of cell death has been through sensitizing hepatoma cells to tumor necrosis factor-related apoptosis inducing ligand (TRAIL) mediated apoptosis. TRAIL at higher doses has been shown to cause toxicity to the healthy cells in addition to cytotoxicity to the cancer cells. Therefore using other agent/drugs in combination with TRAIL could be a viable strategy to induce maximum cytotoxic effects at subtoxic doses of TRAIL. It has been shown that subtoxic doses of GS and TRAIL can generate efficient apoptotic death in hepatoma cells. This GS/TARIL combination has proved to be efficient in inducing the apoptosis by disrupting the disrupting mitochondrial membrane potential resulting in the release of cytochrome C to the cytosol and consequent activation of caspases. In addition GS-mediated ROS generation can lead to upregulation of the death receptor DR5 *via* eIF2α and C/EBP homologous **protein** (**CHOP**). TRAIL binding to DR5 can result in the efficient induction of TRAIL-mediated apoptosis in hepatoma cell [83, 84]. GS has been shown to induce apoptotic cell death in hepatocellular carcinoma cell lines by activating intrinsic mitochondrial pathway [85].

GS was found to have the antifibrotic activity as it mediates reduced activation and survival of hepatic stellate cells (HSCs), which serve as the primary source of the matrix proteins. Accumulation of extracellular matrix has been shown to be involved in liver fibrosis that can lead to cirrhosis of the liver. During cirrhosis, the blood flow through the liver becomes disrupted due to damage in an architectural organization of the liver. Once cirrhosis is developed, the risk of developing liver cancer is significantly increased. It was found that the GS inhibits the growth of immortalized LX-2 HSC cells *via* induction of apoptosis. GS-induced apoptosis in HSC was accompanied by activation of c-Jun N-terminal kinase and mitochondrial apoptotic signaling. GS-induced HSC growth inhibition was also found to involve AKT and adenosine monophosphate-activated protein kinase (AMPK) phosphorylation modifications resulting in the activation of proapoptotic proteins and downregulation of antiapoptotic proteins [83]. GS has also been shown to inhibit NF-κB activation in LX-2 cells where the constitutive activation of this pathway leads to increased growth of these cells [86]. Besides NF-κB activation, increased collagen α1 synthesis and α-smooth muscle actin expression plays a significant role in the growth of HSCs resulting in enhanced liver cirrhosis, and treatment with GS significantly decreased the extent of collagen deposition via inhibiting collagen α1 synthesis and α-smooth muscle actin expression. These findings strongly implicate GS as an antifibrotic agent inhibiting various survival pathways via induction of apoptotic cell death in HSC cells.

Limited studies have also shown that GS induces anticancer activities in lung and ovarian cancers. Treatment of lung and ovarian cancer cell lines with GS resulted in inhibition of cell proliferation and downregulation of cyclin D1 and cdc2 expression leading to inhibition of DNA synthesis. In addition GS treatment also increased the expression of cyclin-dependent kinase inhibitor p21 and p27. Moreover, GS-mediated apoptosis correlated with the activation of JNK, caspase-cascade, accompanied with inhibition of the expression of various anti-apoptotic genes [25].

## Hematological malignancies

Hematological malignancies constitute approximately 6.5% of all cancer incidences worldwide [87]. The primary causes of these liquid cancers are due to defect at the level of bone marrow and lymphatic system [87]. These malignancies are classified into three main groups including leukemia, lymphoma, and multiple myeloma (MM). The anticancer activity of GS in these hematological malignancies is not studied in detail, and this review would enable to pursue further research in this field. Antileukemic effect of GS has been reported by Samudio and colleagues [26] where they examined the anticancer effects of three isomeric pregnadienedione steroids [i.e., cis-GS, trans-GS, and 16-dehydroprogesterone] in HL60 and U937 cells as well as in primary leukemic blasts in culture [26]. They demonstrated that the treatment of HL60 and U937 cells with these compounds prevented cell proliferation via mitochondrial-dependent but caspase-independent apoptosis. All three compounds were shown to induce the generation of ROS which can be one of their mechanisms of cell death. Furthermore, these compounds resulted in the dephosphorylation of constitutive extracellular signal-regulated kinase phosphorylation status in these leukemic cells. Interestingly only cis-GS caused a rapid depletion of glutathione levels as well as oxidation of the mitochondrial phospholipid cardiolipin [26]. The other study carried by Shishodia and colleagues [25] observed that the treatment of leukemia, myeloma and melanoma cell lines with GS resulted in decreased proliferation along with reduced levels of cyclin D1 and cdc2 which inhibited DNA synthesis. They found an increased levels of cyclin-dependent kinase inhibitor p21 and p27 as well as induction of apoptosis by activation of JNK, caspase-cascade, PARP cleavage and downregulation of anti-apoptotic products [25].

## Conclusion

**Fig. 3:**
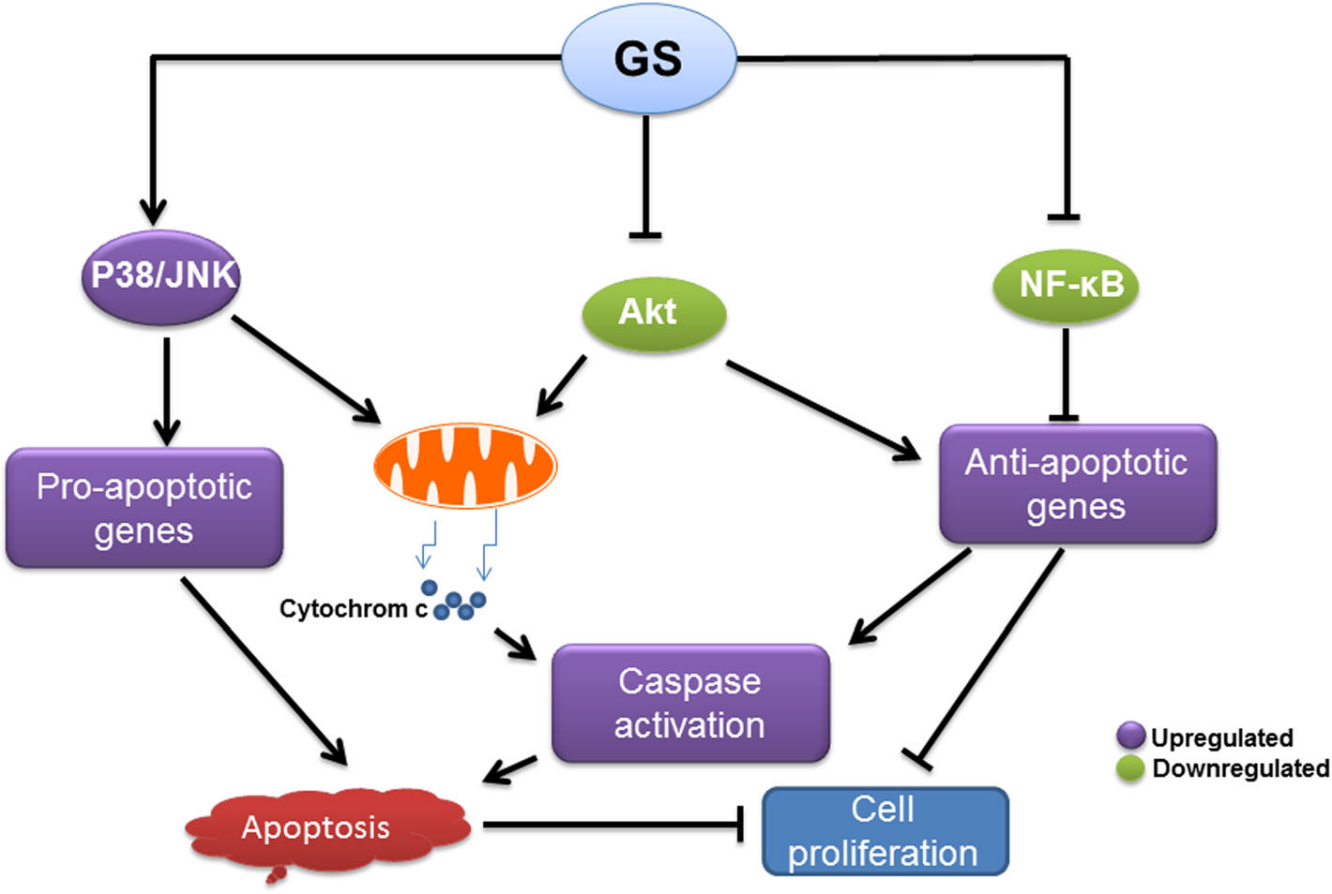
Schematic diagram illustrating the main biological targets of Guggulsterone. The apoptotic effects of guggulsterone are preceded by activation of JNK, suppression of Akt and NF-kB activity. Activation of JNK leads to induction of propapoptic proteins and release of cytochrome c from the mitochondria which in turn activates caspases, resulting in apoptosis. Down regulation of NF-kB activity leads to inhibition of anti apoptotic proteins which in turn activates caspases, resulting in apoptosis and inhibition of proliferation

**Fig. 4:**
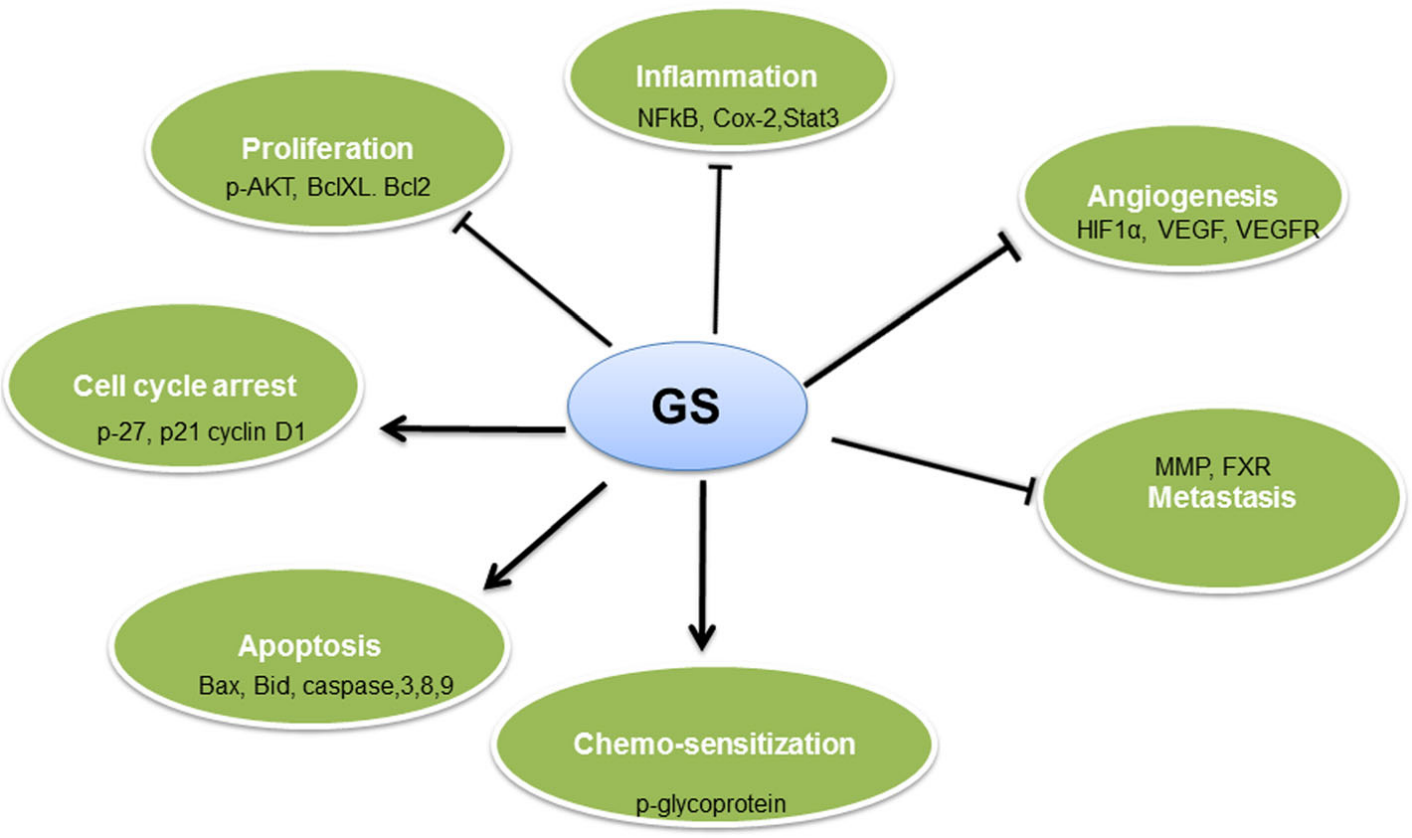
Schematic representation of Guggulsterone mediated effects on various biological processes. i) Abrogating pro-inflammatory signaling by inhibiting activity/expression of IKK-NF-kB, STAT3, COX-2,iNOS, etc. ii) Inhibition of cancer cell proliferation through cell cycle arrest by modulating cyclins, CDKs, etc. iii) Induction of apoptosis of cancerous or transformed cells by modulating expression/activity of caspases, IAPs, Bcl-2 family proteins, etc. iv) Inhibition of angiogenesis by targeting HIF-1a, VEGF, VEGF-R, etc. v) Sensitization of tumor cells to apoptosis induced by chemotherapeutic drugs and reversal of multidrug resistance

There is a growing evidence now that GS is capable of preventing tumor growth and proliferation through activation of pro-apoptotic and inhibition of anti-apoptotic signaling pathways (Fig. 3). GS has been shown to cause effects on the biological function of cells including cell proliferation, angiogenesis, inflammatory response and apoptotic cells death in cancers cells (Fig. 4). GS mediated anticancer effects are due to inhibition of kinase activity of AKT and its downstream targets such as GSK3, FOXO1 and mTOR signaling. GS has been shown to inhibit the activity of many transcription factors such as NF-κB and AP1 that can lead to down regulation of various gene products including c-myc, Bcl-2, COX-2, NOS, Cyclin D1, interleukins and MMP-9. Furthermore, GS affects many growth factor receptors and angiogenic factors such as VEGF, which play a pivotal role in tumor growth, metastasis and angiogenesis. Not only GS-induces apoptosis in cancer cells and inhibits cell proliferation but it is also useful in reducing the cytotoxicity associated with conventional chemotherapeutic agents by sensitizing or causing the additive apoptotic effects. Despite, GS has been extensively studied, the conclusive mechanisms responsible for its anticancer effects are still not fully understood. Outcomes of various preclinical studies suggest that anticancer action of GS and its isomers are due to combined effects on proliferation and invasiveness in cancer cells.

## Future perspective

The rate of incidence of cancer and resulted deaths are alarming around the world despite the accessibility of various therapeutic options for cancer patients. Most modern medicines currently available for treating cancer are synthetic, mono-targeted, very expensive, less efficient and often possess severe side effects. Therefore, there is a critical need to develop alternative drugs for the management of cancer.

Phytochemicals, a family of naturally occurring compounds including polyphenols, carotenoids and steroids have been demonstrated to have anticancer activities against a variety of cancers both in vitro and in vivo. Among these compounds, guggulsterone (GS), a steroid by nature recently has attracted the attention of cancer researchers and investigators for its anticancer potentials. GS has been shown to induce efficient apoptotic cell death in a variety of cancer cells. Interestingly, no apoptotic death was seen in healthy cells. A number of studies further showed significant cellular changes induced by GS *via* modulating distinct signaling molecules involved in carcinogen detoxification, cell proliferation, angiogenesis, metastasis, multi-drug resistance, etc. In addition, GS has been shown to sensitize the effects of chemotherapeutic drugs in in vitro system. These anticancer activities in preclinical settings are potentially beneficial in treating cancer. Further studies directed towards target identification and pathway analysis could pave the way for the addition of GS to the management of anticancer therapy. Despite the availability of extensive preclinical data on anticancer potentials of GS, there is a lack of studies accounting for its safety and bioavailability, which needs to be pursued. Safety of long-term use of GS needs to be evaluated in clinical settings, but appears to be devoid of acute, subacute, chronic toxicity in rats, dogs, and monkeys; no mutagenic or teratogenic effects have been reported. Ayurvedic system of medicine describes GS as safe and efficient medicine; however, it should be used cautiously in combination with prescribed drugs as it may modulate the activity of drug metabolizing enzymes. As soon as a consensus on its safety and bioavailability emerges, a planned Phase I clinical trials should be perused to validate its usefulness as anticancer agents and must be prioritized for different site-specific cancers. The outcome of these studies may lead to development of new and efficient therapeutic trategies for the management of cancer.

## Abbreviations

AMPK: Adenosine monophosphate-activated protein kinase
DSS: Dextran sulfate sodium
ERK1/2: Extracellular signal-regulated kinase 1/2
FXR: Farnesoid X receptor
GL: Guggul lipid
GS: Guggulsterone
HNSCCC: Squamous cell carcinoma
HSCs: Hepatic stellate cells
IBD: Inflammatory bowel disease
IR: Ionizing radiation
JNK: c-Jun NH(2)-terminal kinase
NF-kB: Nuclear factor-kB
PARP: Poly-ADP-ribose phosphate
PrEC: Prostate epithelial cell line
TRAIL: Tumor necrosis factor-related apoptosis inducing ligand
VEGF: Vascular endothelial growth factor
